# Genetic structure and relatedness of juvenile sicklefin lemon shark (*Negaprion acutidens*) at Dongsha Island

**DOI:** 10.1038/s41598-023-28186-y

**Published:** 2023-01-18

**Authors:** Shang Yin Vanson Liu, Yu-Yun Chen, Chi Cheng

**Affiliations:** 1 Department of Marine Biotechnology and Resources, National Sun Yat-sen University, Kaohsiung, Taiwan; 2 Doctoral Degree Program in Marine Biotechnology, National Sun Yat-sen University, Kaohsiung, Taiwan; 3 Graduate Institute of Natural Products College of Pharmacy, Kaohsiung Medical University, Kaohsiung, Taiwan; 4 Division of Natural Science, General Education Center, Aletheia University, New Taipei City, Taiwan

**Keywords:** Population genetics, Molecular ecology

## Abstract

*Negaprion acutidens* (sicklefin lemon shark) is distributed in the Indo-Pacific and in close association with coral reefs. Under the protection of the Dongsha Atoll National Park, a small but well-established juvenile population of *N. acutidens* inhabiting coastal areas of Dongsha Island was recently observed to display site fidelity by using acoustic telemetry. This study was designed to reveal the fine scale genetic structure and relatedness within and among 5 juvenile shark cohorts inhabiting 3 sampling sites at Dongsha Island. A total 188 juveniles were caught and sampled between 2016 and 2017, and genotyped with twelve loci. They were assigned to 5 year cohorts (2013–2017) based on the body length and date they were caught, also assigned to 3 sites based on where they were caught. Among five cohorts, the percentage of unrelated pairs within a cohort is more than 62% in average, suggesting a potential high mortality during their early life stage. The results of Fst and assignment testing showed that there was no significant genetic structure between sites and cohorts indicating that there was no fine scale genetic structure, even though the juveniles possessed strong site fidelity. A small effective population size (Ne) was detected (Ne = 86.7) which indicates the presence of a potentially isolated and vulnerable population at Dongsha. These results provide the genetic diversity as a baseline for future management and conservation of *N. acutidens* in the South China Sea.

## Introduction

Sharks are the most endangered group of marine animals due to their vulnerability to fishing pressure^1,2^. Information on abundance and distribution of most of the shark species is relatively scarce, with deficient data occurring in 45% of the species, according to red list assessments of the International Union for Conservation of Nature (IUCN)^3^. According to the report of Food and Agriculture Organization of the United Nations (FAO), global landing of chondrichthyans increased since 1950s, reaching a maximum in 2000 (888,000 mt) before declining. However, global landings data are likely to represent a gross underestimation of actual landings due to illegal fishing. The high value of shark fins has led to an increase in illegal shark finning, resulting in an increase in the number of discarded bodies which often go unreported^4^. In Southeast Asia, the shark catch records are often inadequate and incomplete^5^. Lam and Sadovy^6^ suggested all known shark fisheries in the South China Sea collapsed between the 1970s and the 1990s and of the 109 species historically present in the South China Sea, only 18 species were recorded in their fish market surveys between 2006 and 2008. Additionally, sharks observed in the markets are almost all subadults, which is a sign of overfishing and inappropriate management. Therefore, there is an urgent need to enhance conservation efforts and implement management plans for these rapidly declining species on either a regional or global scale.

The genus *Negaprion* is comprised of two species, including *Negaprion brevirostris* (lemon shark) an inhabitant of shallow, inshore waters throughout the western Atlantic also occurring in eastern Pacific and eastern Atlantic and *Negaprion acutidens* (sicklefin lemon shark), which is found in similar habitats across the Indo-Pacific region. Both species are associated with reefs. The former species has been overexploited^7^ and the latter species is locally extinct in India and Thailand, endangered in Southeast Asia, and considered vulnerable throughout its range [IUCN Redlist; www.iucnredlist.org]. Both species have shown strong site fidelity^8,9^. The lemon shark, *N. brevirostris,* is one of the most well-studied elasmobranch species in the world. The juvenile phase has been found to utilize shallow coastal environments (lagoons) as nursery habitat. Strong site fidelity has been revealed by genetic and acoustic telemetry in different regions of the western Atlantic^8,10,11^. Additionally, female lemon sharks exhibit natal philopatry to one of these sites (Bimini, Bahamas)^12^. This understanding of the connectivity, behavior, and spatial ecology of elasmobranch specie is crucial for the development of effective conservation strategy and management plans.

In contrast, little is known about the movements, behavior, and reproductive biology of the sicklefin lemon shark (*N. acutidens*), largely because of its overall scarcity due to recent population decline [IUCN Redlist; www.iucnredlist.org]. This species has been extirpated in India, Thailand, and Bali (Indonesia) due to overexploitation, but it remains relatively abundant in Indian Ocean and coastal areas of Australia^9,13^. Schultz et al.^14^ conducted the first genetic study on sicklefin lemon shark in the region between western Australia to central Pacific showing a minor genetic structure which may be due to the isolation by oceanic distance in between, the juveniles exhibit site fidelity by a narrow home range, similar to the closely related lemon shark^9,15,16^. There are only a few records of the occurrence of sicklefin lemon shark in the South China Sea^17,18^. The Dongsha Island (20° 40′ 34.5″N 116° 49′ 31.7″ E) is a remote island, part of Dongsha Atoll which is located at the northern boundary of the South China Sea approximately 450 km from Taiwan. The island is 2.8-km long and 0.865-km wide with an embedded lagoon (64 ha). A recent survey of the fish composition in the embedded lagoon showed that juvenile *N. acutidens* are among the three most abundant fish taxa found in the lagoon^18^. Like other coastal sharks, sicklefin lemon sharks, use shallow coastal habitats such as seagrass beds and lagoons as nursery grounds, then gradually expand their territory as they grow older^9^. In addition, site fidelity has been observed in not only juveniles but also resident adults of *N. acutidens*^15,19^. Based on the results of long-term acoustic telemetry data for studying the movement of juveniles inhabit around Dongsha Island, juveniles inhabit different sites around the island (i.e., north shore and small lagoon) exhibited strong site fidelity (Chen unpublished data). These behaviors related to fidelity for specific breeding and nursery areas may result in genetic structure despite the high mobility of adults on a relatively large scale (100 s–1000 s km) ^20,21^. This aggregation of juvenile sharks indicate Dongsha Island may represent as an essential habitat for sicklefin lemon shark to reproduce or as nursing ground like other sharks^22,23^ and have disproportionate management implications for shark conservation^24^. In the present study, in order to reveal the spatial and temporal genetic structure of juvenile *N. acutidens* at Dongsha Island, we developed eight novel microsatellite loci in combination with four previously published microsatellite loci to determine the genotype of 188 juveniles collected during 2016 and 2017. This information was used to determine whether the site fidelity affects spatial genetic structure around the Island.

## Materials and methods

### Sampling

**Figure 1:**
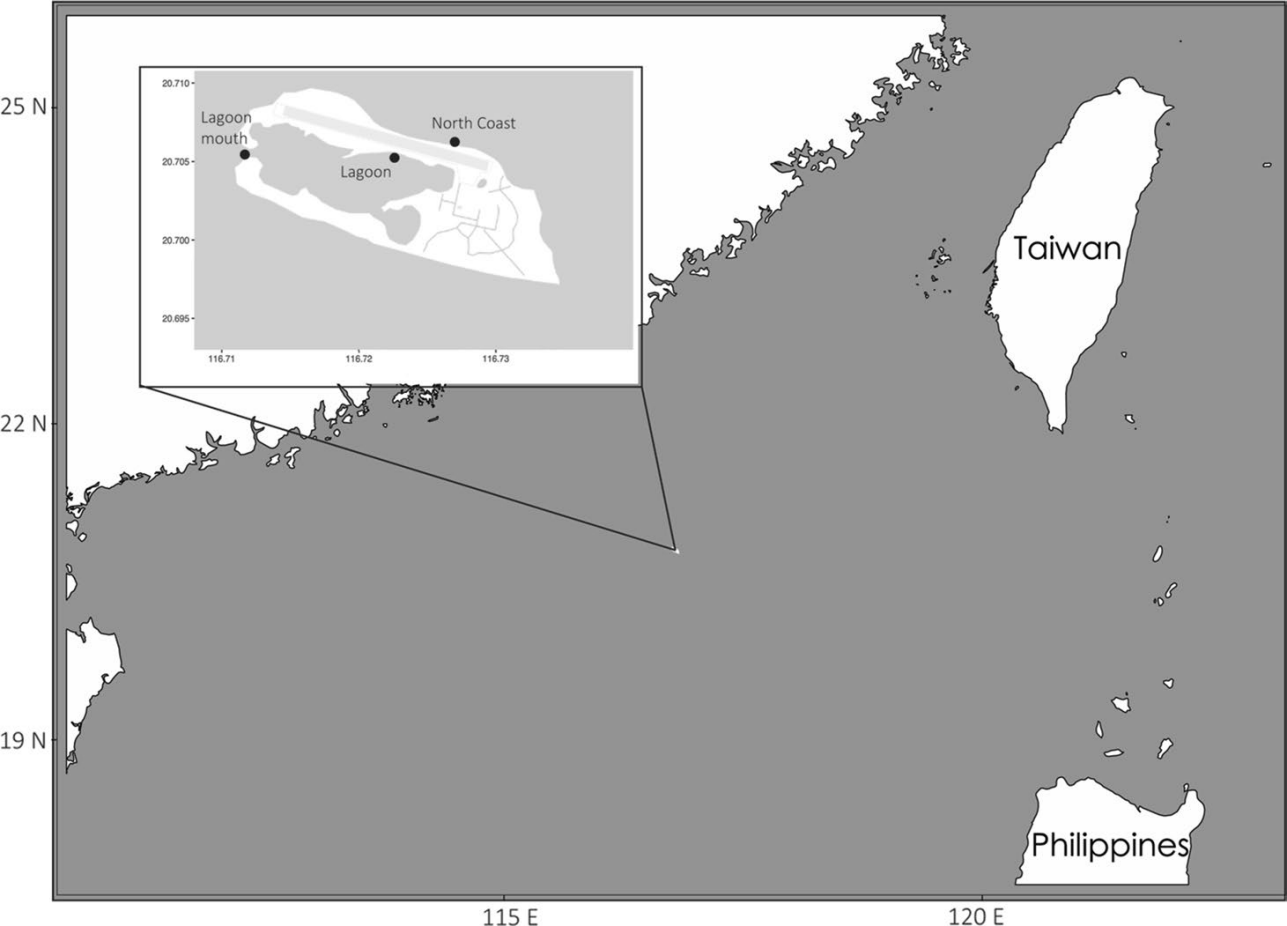
Sampling map of this study which generated by R package ggmap version 3.0.1 (https://github.com/dkahle/ggmap) and ggOceanMaps version 1.3.7 (https://mikkovihtakari.github.io/ggOceanMaps/).

Pot on traps designed for catching juvenile shark were deployed in the lagoon, at the lagoon mouth, and in shallow seagrass beds along the northern coast of island during 2013–2017 (Fig. 1). A total of 188 juveniles were caught and total length and weight of each individual were measured. Juveniles were tagged with either t-bar tag or chip along with an acoustic tag before release, a small tissue was taken and preserved in 95% EtOH immediately during this process.

### Cohort assignment

In 2013 the Marine National Park headquarters funded and initiated a long-term monitoring project on the dispersal pattern of juvenile sicklefin lemon shark around Dongsha Island. In order to assign the juveniles used in the present study to different year cohorts we selected 55 events of capture-recapture records from 2014 to 2017. Length data in which the time lapse between the 1st and last observations was more than 50 days were used to estimate the average growth rate (cm/month) of juveniles (Table S1). Additionally, we extracted the 1^st^ observation events (n = 246) while we observed the caught juveniles with open or recently closed umbilical scar between April and May (adult female sharks give birth during this period on Dongsha Island) from 2013 to 2017 to estimate the average total length of newborn pup (Table S2). Using the information on growth rate and average total length of each newborn pup we estimated the age of captured juveniles in months. We then used the capture date to determine which year each juvenile was born to define its year cohort based on the following formula:

$${\text{The age of captured juvenile in months}} = {\text{TL}} - {\text{TL}}/{\text{GR}}$$

TL = Total length of captured juvenile, TL = Average total length of newborn pup, GR = Average growth rate of juvenile (cm/month).

### Microsatellite isolation and analyses

Genomic DNA was extracted from muscle tissue using commercial DNA extraction kits (Genomic DNA Mini Kit, Geneaid Biotech, Taiwan). Through the raw sequences from a paired-end Illumina Miseq sequencing run, 41,546 contiqs (> 500 bp) were obtained after QC and assembly. A total of 84 sequences with different microsatellite motifs were isolated and primers were designed by Primer 3 (version 0.4.0) including 78 dinucleotide, 2 complex-nucleotide, 2 tetra-nucleotide and 2 hexa-nucleotide repeats. all quality filtered and assembled reads were used for screening repeated motifs following the methods described in ^25^. In total, eight novel microsatellite loci (Table 1) were developed from shotgun sequencing. A PCR gradient test (annealing temperature at 50 °C, 52 °C, 54 °C, 56 °C, 58 °C and 60 °C) was used to determine the best annealing temperature for each locus. Four published loci were applied for the final genotyping process. Fragments were amplified in 25 μL reactions in an ABI Veriti gradient thermocycler under the following program: 95 °C for 3 min, followed by 30 cycles of denaturation at 94 °C for 30 s, annealing at the best annealing temperature we got from the gradient test for each locus for 30 s, and extension at 72 °C for 30 s with a final extension at 72 °C for 1 min. Each reaction contained 30 ng DNA, 12.5 ul Taq DNA Polymerase 2X master Mix RED (0.4 mM each dNTPs, 15 mM MgCl, 0.2 unit of Ampliqon DNA polymerase) (Ampliqon, Denmark) and each of primers (200 nM). The forward primers of 12 loci were label with FAM or TAMRA at 5’for genotyping with 500 LIZ Size Standard on ABI 3730 sequencer. GeneMapper® Software v. 4.1 (Applied Biosystems, USA) was used to detect allele sizes through the peak pattern of fluorescent value. Parameters including allele frequencies, mean number of alleles (NA), observed (H_O_), expected (H_E_) heterozygosities and Fst were estimated using Microsatellite Analyzer (MSA) ^26^. An exact test on genotype counts was done to test for significant deviations from Hardy–Weinberg equilibrium by GENEPOP. MICRO-CHECKER, version 2.2.3 ^27^ was used to check for the possible occurrence of null alleles and allelic dropout. STRUCTURE, version 2.2 ^28,29^ was used to infer population structure and assign individuals to clusters based on microsatellite genotype. The software was run using the admixture model that assumes that all individuals are potentially of mixed ancestry, and assigns each individual to a designated population (of K potential populations) with a partial probability. Ten independent runs, incorporating a burn-in of 1 000 000 Markov chain-Monte Carlo iterations followed by 1 000 000 replicates of data collection. For data set of location, we set K = 1 to 3 because we assigned 188 juveniles to 3 populations according to where they were caught (north shore, mouth of lagoon and inner lagoon). For cohort data sets, we set K = 1 to 5 because we assigned 188 juveniles (we excluded one individual which was the only one assigned to year 2012) to 5 cohorts (2013, 2014, 2015, 2016 and 2017). Structure Harvester 0.6.94^30^ was used based on the ΔK obtained through each run to determine the most likely number of clusters for each data set. CLUMPP^31^ was used to summarize the results of replicates under the best K-value. To further understand the clustering patterns, genetic distance-based (Pairwise Nei genetic distance) principal coordinate analysis (PCoA) was carried out using GENALEX 6.5^32^. Analysis of molecular variance (AMOVA) was further performed by separating 188 individuals into two groups based on the PCoA result with ‘poppr’ package^33^ in R.

**Table 1:** Information of microsatellite loci used in this study. (Na = number of allele, Ho: observed herterozygosity, He = expected herterozygosity).

Loci	Motif	Primer-forward	Primer-reverse	Na	Ho	He	Reference
LS11	(AC)33	5′-CCAGGAGAGAAGCATCTCACAG-3′	5′-TGTCATTAGGATTTGCAGCC-3′	16	0.596	0.678	^47^
LS24	(AC)12	5′-GGATGTGTTAGTGAGGTGGTGAGTG-3′	5′-AGGGCAGAGACAGCAGGGAATATC-3′	4	0.266	0.279	^47^
LS54	(CT)10(CA)8	5′-TTGGAAACCGTGGAGGTGAA-3′	5′-GGGGAAAAAGAACTGGGACTAATCC-3′	5	0.357	0.308	^48^
CPl90	(AC)_24_	5′-GTTGTTGCCTTGTCTTTCAATCG-3′	5′-TGTGTCACTGTGTCTCTGTGTGCC-3′	10	0.36	0.675	^49^
NA08	(TG)28	5′-CCTCCAGCGCACTCATCTTT-3′	5′-GGGTATTATTGCTGCACGGC-3′	17	0.799	0.804	present study
NA10	(AATT)7	5′-GAGTCCCGGGCTAACTTCAC-3′	5′-GGTAGGTACTCGGGTCACCT-3′	6	0.487	0.541	present study
NA12	(TG)14	5′-GCGTGCGTATGTGTGTGTG-3′	5′-GCAGGTTGGACAGAAGACCA-3′	4	0.275	0.364	present study
NA13	(CA)21	5′-GAGACGATCCTGTGCCGTAA-3′	5′-ATGTGAGGGGACATGGCAAG-3′	22	0.724	0.76	present study
NA14	(GT)28	5′-CACTCAAGGAGCTGATGACCA-3′	5′-AGTGCCCTGAGATTGGATGTG-3′	15	0.505	0.709	present study
NA16	(TA)15	5′-TGGGGTTTCCATTCCCAATT-3′	5′-GGAGCTCCTGGACTTTGACC-3′	14	0.671	0.745	present study
NA19	(GT)14	5′-GGTGTGTGGAATGGTGCTTC-3′	5′-AGCGTTCCATGTTTGTGGGA-3′	3	0.01	0.259	present study
NA20	(TA)16	5′-ACGGCAGAGAATGTAGCTCT-3′	5′-AGAAATCCAGTAATGACGTTGGT-3′	5	0.195	0.179	present study

Maximum likelihood estimates of pairwise relatedness coefficients and genealogical relationships were calculated with the software ML-RELATE^34^, computing 5000 iterations for each year cohort. The program calculates the maximum likelihood relationship between individual pairs. It determines which of the following yield the greatest likelihood: parent offspring (PO), full-sibling (FS), half sibling (HS) and unrelated (U) categories. Meanwhile, COLONY version 2.0.6.6 was also used with marker type error rate as 0.001 for each locus, both male and female were polygamy to determine whether the relationship of a pair of juveniles belongs to one of three possible candidate relationships, including full-sibling, half-sibling and non-sibling. In order to quantify the effective population size of the *N. acutidens* inhabiting the waters of Dongsha, we used NeEstimator v2.1^35^ based on linkage disequilibrium (LD) for two allele frequency thresholds (0.02 and 0.05) and one without any constraints.

All field work was carried on in accordance with the relevant guidelines and regulations under Marine National Park Headquarters permit No. 1051000471 and No. 106000672. This study was approved by the Institutional Animal Care and Use Committee (IACUC) at National Sun Yat-sen University and conducted the experiments following the IACUC guidelines. All methods are reported in accordance with ARRIVE guidelines.

## Results

The observed heterozygosity and expected heterozygosity of each locus in each population were given in the Table S3(Ho: 0–0.9, He: 0.19–0.9).

### Cohort assignment

The average juvenile growth rate was 0.868 cm/month, and the average total length of newborn pup was 67.47 cm. Ten juveniles were assigned to the 2013 cohort, 24 juveniles were assigned to 2014, 85 juveniles were assigned to 2015, 45 juveniles were assigned to 2016, and remaining 24 juveniles were assigned to 2017 (Table S4).

Among them, we developed, characterized and applied 8 novel polymorphic microsatellite loci including seven dinucleotide loci and one tetra-nucleotide loci, with another 4 published loci (Table 1) to genotype 188 juvenile sicklefin lemon sharks. Across 12 loci among 188 samples, Na ranged from 3 to 22, Ho ranged from 0.01 to 0.799 and He ranged from 0.179 to 0.804. Except for CP190, NA14 and NA19, the loci showed no significant heterozygosity deficiency. The presence of null allele was also found in these three loci. We therefore decided not to include the data of these three loci for further analyses. For the remaining nine loci, no significant deviation from Hardy–Weinberg was observed in the global population (*P* > 0.05).

**Figure 2:**
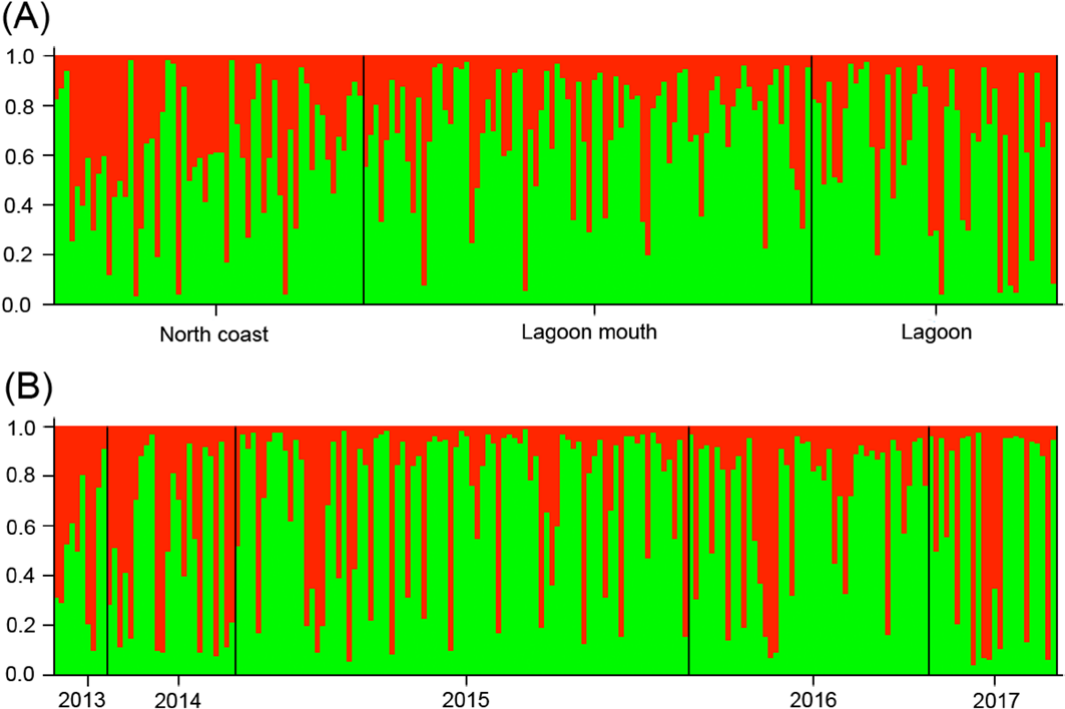
Assignment test performed by STRCTURE under K = 2 scenario.

**Figure 3:**
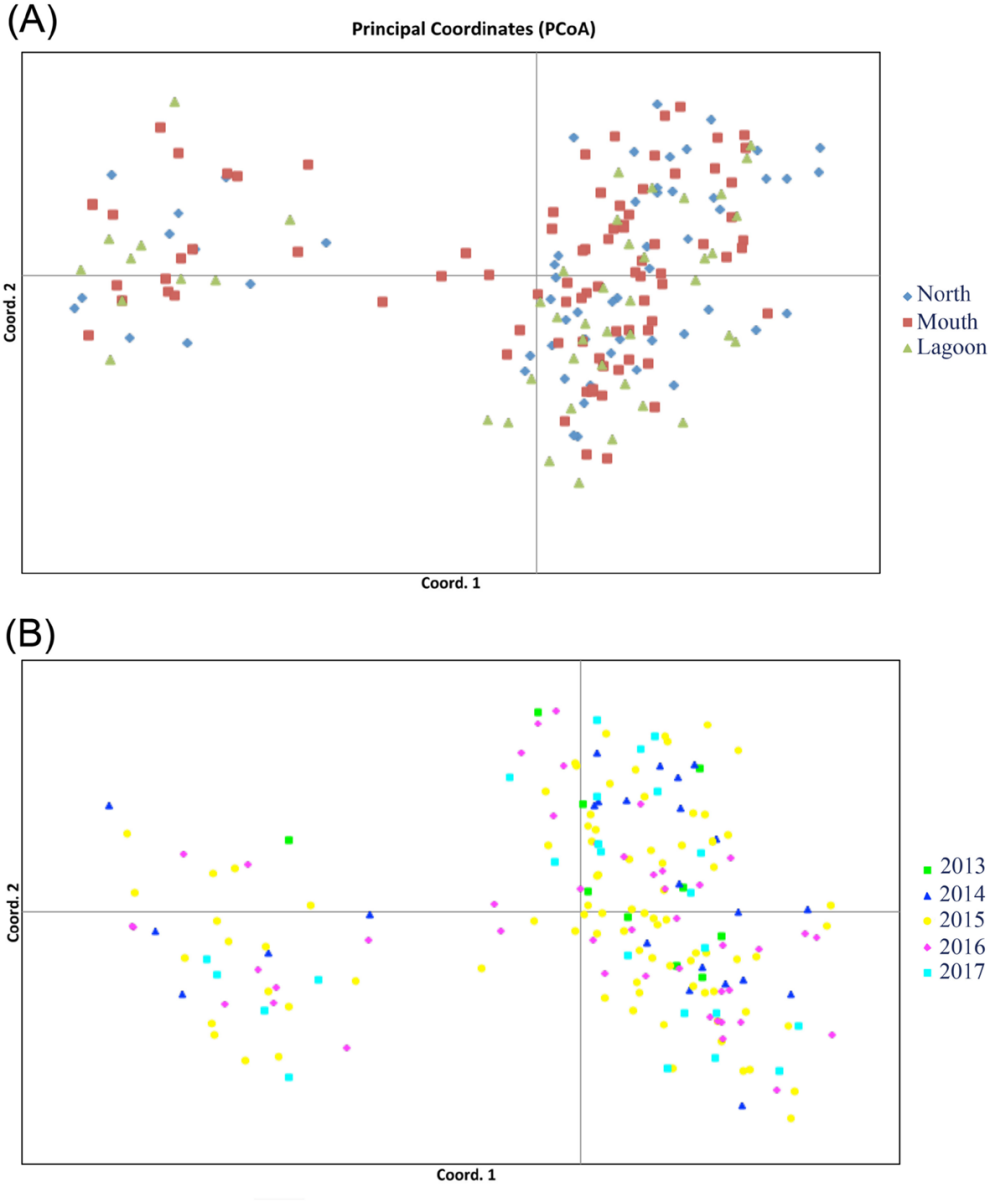
Principal coordinate analysis (PCoA) on individual microsatellite genotypes. A) PCoA based on 9 microsatellite loci from 188 juveniles assigned to 3 locations. B) PCoA based on 188 juveniles assigned to 5 cohorts. Each symbol represents a unique individual with symbol color and shape denoting different assignment design.

The results of STRUCTURE showed that K = 2 has the highest delta K for both location and year cohort data sets. The bar charts represent the results of the assignment test while K = 2 (Fig. 1). Both bar charts showed no obvious population subdivision (Fig. 2). The results of pairwise Fst test (Tables S5 and S6) showed a similar pattern that of the assignment test. No genetic structure was found among three sampling locations (north shore, mouth of lagoon and inner lagoon) and 5 cohorts (2013–2017). The results of principal coordinate analysis showed a clear pattern that the juveniles used in this study were divided into two groups (pc1 + pc2 = 15.49%), showing no relationship with geographic location or year cohorts (Fig. 3A,B). The result of AMOVA indicated a significant genetic structure between two populations (Fct = 0.1341, *P* < 0.01, 13.4% of variance). The estimated effective population size (Ne) by the linkage disequilibrium method under the allele frequency thresholds of 0.02, 0.05 and no constraint were 86.7 (95% CI = 75.3–100.6), 87.9 (95% CI = 71.7–109.5) and 199.9 (95% CI = 170.2–239.1), respectively.

**Figure 4:**
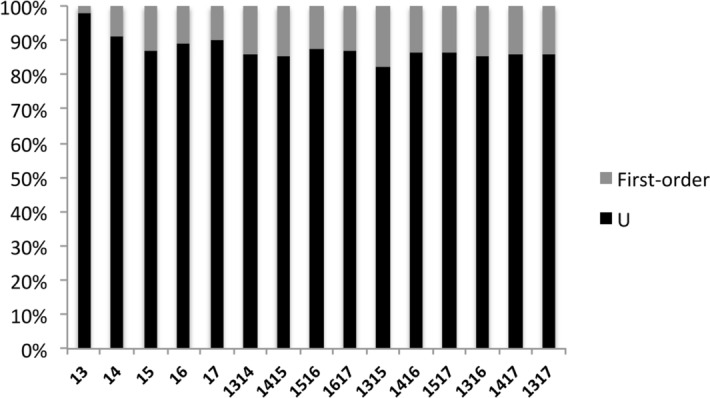
Bar charts of genetic relatedness based on pairwise genetic relatedness in each and between cohorts. (U = unrelated, First-order = PO + FS + HS).

Since the samples we used in this study are all juveniles, we didn't detect any parent-offspring (PO) relationship in each cohort. The first order genetic relationships (PO, FS and HS) accounted for 2%, 10%, 13%, 11% and 10% of all pairwise relationships in year 2013, 2014, 2015, 2016 and 2017, respectively (Fig. 4). Generally, the results derived from COLONY were mostly concordant with the pairwise relationship obtained from ML-RELATE (mostly belong to non-sibling relationship), except in 2013 cohort which were dominated by half-sibling (80%) (Figure S2). In contrast, the first order genetic relationship between cohorts was higher between cohorts and significantly higher than within year cohorts (*P* < 0.05, unpaired t-test). Among these cohorts, an average of 91% of the pairwise relationships were unrelated (Fig. 4).

## Discussion

### Spatiotemporal genetic structure and relatedness

Even previous studies showed that the juvenile *N. acutidens* has a relatively restricted home range at St. Joseph Atoll in the Indian Ocean^9^, northern Australia (< 1.9 km^2^) ^36^ and even smaller in western Australia^16^ (0.6 ± 0.04 km^2^). And fine spatial side fidelity was also detected between three sites (North coast, Lagoon mouth and Lagoon) where juveniles were collected in the present study. The results of pairwise Fst test showed no genetic structure among these three sites which suggest that this behavior may not affect the fine scale genetic structure of juvenile *N. acutidens* inhabit in the water of Dongsha Island. Surprisingly, we observed a clear genetic partition based on the PCoA plot (Fig. 3), and the result of AMOVA test also support this finding. As female of *N. acutidens* has been documented to have strong philopatric behavior by using genetic and tagging methods^15,36^. With the results of pairwise relationship analysis, a significant higher first order genetic relationship was found between year cohorts than within a given year cohort. This indicates the mature females may consider Dongsha Island as natal grounds, returning to the same place to give birth every one or two years, as suggested by^37^. Unfortunately, the adult *N. acutidens* are rarely spotted and caught in the shallow water around Dongsha Island because they generally use a deeper water as habitat, only approaching the shallow water for pupping (Chen personal observation). Collection of tissue samples from adults could allow us to verify our findings by reconstructing the pedigree between adults and juveniles inhabiting the Dongsha. Additionally, further study using popup satellite tagging is needed to better understand adult movement in the Dongsha Atoll.

Our results of relationship test also showed that an average of 91% and 62% of the pairwise relationships were unrelated by ML-RELATE and COLONY, respectively. This evidence may also suggest that we observed an outcross population in Dongsha Island. Previous study documented in French Polynesia showed two times higher first-order genetic relationship in Moorea than in Dongsha. Although the Moorea study focused on only 40 mature sharks and our study focused on juveniles only (188 individuals), we suggest the genetic network of different geographic populations may vary. Additionally, *N. acutidens* is placentally viviparous, producing 1–13 (mean = 9.3) pups per litter^38,39^. With an extremely low first order genetic relationship found in each year cohort and high sampling intensity of the capture-recapture experiment (monthly), we suggest that the natural mortality rate of newborn sicklefin lemon sharks may be very high in Dongsha population, resulting in survival of only a few pups per litter to reach the juvenile stage instead of causing by incomplete sampling.

### Effective population size

The Dongsha Island is the only known nursery ground for *N. acutidens* in the South China Sea with high juvenile abundance in the shallow habitats^18^, (Chen unpublished data) and it is located far from other potential habitats such as West Luzon coast (500 km), Spratly Islands (1160 km) and Paracel Islands (670 km). Although previous study has showed that *N. acutidens* could potentially travel up 300 km between Moorea and Bora Bora^15^. Unlike French Polynesia which contains many islands adjacent to each other to serve as stepping-stones to support their seasonal movement, Dongsha Island is relatively isolated. The migratory path to West Luzon coast and Spratly Islands crosses more than 500 km deep ocean basin without any island in between, therefore, the only possible migratory path may follow the continental shelf toward west to reach Hanan Island or Paracel Islands and the travel distance is more than 650 km. Under the protection of the Dongsha Atoll National Park, the nursery ground at Dongsha Island may have acted as the population source for other local populations and for the whole northern South China Sea area since 2013. It is important to improve understanding of the effective population size (Ne) of this putative source population for purposes of assessing extinction risk and improving conservation management of this threatened species^40–42^. Frankham et al.^43^ proposed threshold requirements of Ne ≥ 100 to avoid inbreeding depression and Ne ≥ 1000 to retained evolutionary potential. Considering these recommendations, the estimated Ne of *N. acutidens* (86.7, 95% CI = 75.3–100.6, Pcrit = 0.02) is insufficient for short-term and long-term health of this species, perhaps representing an isolated population at Dongsha Island^44^. However, the Ne estimated by microsatellite may lack accuracy and precision when derived from insufficient numbers of samples and loci^45^. Dudgeon and Ovenden^46^ provided guidelines for establishing the minimum numbers of samples (91 samples or 20% of the census population) at least 10 loci in order to obtain adequate precision and finite values for estimating Ne of the zebra shark, *Stegostoma fasciatum*. In the present study, we used 188 samples with 9 microsatellite loci, which should be enough to obtain a fairly robust Ne estimation under the threshold provided by^46^. Therefore, the Ne value of the present study suggest that the Dongsha Island population is relatively isolated with potential to suffer inbreeding depression in the future.

In conclusion, the coupling of genetic analyses with long-term demographic experiments is crucial to improving our understanding of the movement patterns of the *N. acutidens* in Dongsha. In present study, we have provided robust evidence that a well-established population of *N. acutidens* inhabits Dongsha waters, using the Dongsha Island as nursery ground. We found that the site fidelity observed in juveniles is not affecting the fine scale genetic structure, and no genetic structure was found among cohorts. Surprisingly, the principal components analysis showed that the population in Dongsha might be comprised of individuals derived from two breeding families. Additionally, the small Ne value that we obtained indicates the juvenile *N. acutidens* found in Dongsha may represent a small, isolated and outcross population. These results provide useful insights for future management and conservation of the sicklefin lemon shark in the South China Sea.

## Supplementary Information

Supplementary Information.

## Data Availability

The dataset of 12 microsatellite loci generated and/or analysed during the current study are publicly available in Figshare (https://doi.org/10.6084/m9.figshare.20436468.v1).
