# Supplementary material for: Genetic structure and relatedness of juvenile sicklefin lemon shark (Negaprion acutidens) at Dongsha Island
Source: Sci Rep. 2023 Jan 18;13:988. doi: 10.1038/s41598-023-28186-y (PMC9849347; doi:10.1038/s41598-023-28186-y)
Supplement: Supplementary file 1 — Supplementary Information. [file 41598_2023_28186_MOESM1_ESM.docx]

Figure S1 Plot of Delta K according to K=5 (5 cohorts).


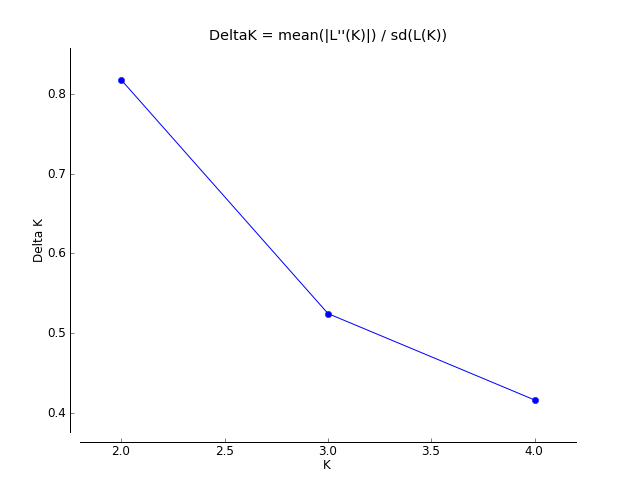


Figure S2 Bar charts of genetic relatedness based on pairwise genetic relatedness across different year cohorts revealed by COLOLY.

Table S1 Fifty-five events of capture recapture record from 2014-2017.

| Tag No. |  | capture date | TL | Duration (day) | cm/month |
| --- | --- | --- | --- | --- | --- |
| 1933 | 1st | 140624 | 72 | 640 | 1.2421875 |
|  | last | 160315 | 98.5 |  |  |
| 1063 | 1st | 160316 | 70 | 80 | 0.1875 |
|  | last | 160604 | 70.5 |  |  |
| 1064 | 1st | 160313 | 83 | 47 | 1.91489362 |
|  | last | 160429 | 86 |  |  |
| 1242 | 1st | 150519 | 69.5 | 350 | 0.21428571 |
|  | last | 160503 | 72 |  |  |
| 1089 | 1st | 160510 | 74.5 | 209 | 0.50239234 |
|  | last | 161205 | 78 |  |  |
| 1076 | 1st | 160502 | 64 | 161 | 0.1863354 |
|  | last | 161010 | 65 |  |  |
| 1269 | 1st | 151013 | 71 | 359 | 1.62952646 |
|  | last | 161006 | 90.5 |  |  |
| 6020 | 1st | 161009 | 72 | 92 | 0.32608696 |
|  | last | 160604 | 70 |  |  |
| 1099 | 1st | 160904 | 71 | 91 | 0.32967033 |
|  | last | 160604 | 76.5 |  |  |
| 1097 | 1st | 160903 | 77.5 | 122 | 0.86065574 |
|  | last | 160504 | 65.5 |  |  |
| 1080 | 1st | 160903 | 69 | 541 | 0.94269871 |
|  | last | 171127 | 84.5 |  |  |
| 1096 | 1st | 160604 | 73.5 | 129 | 1.04651163 |
|  | last | 161011 | 78 |  |  |
| 1090 | 1st | 160502 | 64 | 161 | 0.1863354 |
|  | last | 161010 | 65 |  |  |
| 1076 | 1st | 151009 | 71 | 333 | 1.48648649 |
|  | last | 160906 | 87.5 |  |  |
| 1280 | 1st | 151011 | 71 | 361 | 1.62049862 |
|  | last | 161006 | 90.5 |  |  |
| 1269 | 1st | 160604 | 66 | 316 | 0.47468354 |
|  | last | 170416 | 71 |  |  |
| 1091 | 1st | 161009 | 63.5 | 57 | 0.78947368 |
|  | last | 161205 | 65 |  |  |
| 1094 | 1st | 161009 | 72 | 97 | 0.6185567 |
|  | last | 170114 | 74 |  |  |
| 6032 | 1st | 160313 | 71 | 212 | 1.13207547 |
|  | last | 161011 | 79 |  |  |
| 1050 | 1st | 150719 | 71.5 | 474 | 1.80379747 |
|  | last | 161104 | 100 |  |  |
| 1266 | 1st | 160429 | 69 | 190 | 0.39473684 |
|  | last | 161105 | 71.5 |  |  |
| 1092 | 1st | 161009 | 64 | 57 | 0.52631579 |
|  | last | 161205 | 65 |  |  |
| 1067 | 1st | 161009 | 72 | 97 | 0.6185567 |
|  | last | 170114 | 74 |  |  |
| 6041 | 1st | 161009 | 61 | 98 | 0.15306122 |
|  | last | 170115 | 61.5 |  |  |
| 6033 | 1st | 161105 | 72 | 309 | 1.74757282 |
|  | last | 170910 | 90 |  |  |
| 6047 | 1st | 161009 | 66 | 199 | 0.15075377 |
|  | last | 170426 | 67 |  |  |
| 6064 | 1st | 161009 | 69 | 97 | 0.15463918 |
|  | last | 170114 | 69.5 |  |  |
| 6091 | 1st | 161009 | 70 | 97 | 0.30927835 |
|  | last | 170114 | 71 |  |  |
| 6034 | 1st | 161203 | 66 | 206 | 0.72815534 |
|  | last | 170627 | 71 |  |  |
| 6042 | 1st | 151106 | 71 | 436 | 0.82568807 |
|  | last | 170115 | 83 |  |  |
| 6070 | 1st | 160904 | 70.5 | 133 | 0.11278196 |
|  | last | 170115 | 71 |  |  |
| 6024 | 1st | 161105 | 71.5 | 71 | 0.42253521 |
|  | last | 170115 | 72.5 |  |  |
| 6048 | 1st | 160905 | 69 | 230 | 0.39130435 |
|  | last | 170423 | 72 |  |  |
| 6097 | 1st | 170420 | 76 | 69 | 2.17391304 |
|  | last | 170628 | 81 |  |  |
| 6026 | 1st | 170416 | 68 | 155 | 0.96774194 |
|  | last | 170918 | 73 |  |  |
| 4914 | 1st | 170624 | 69 | 80 | 0.5625 |
|  | last | 170912 | 70.5 |  |  |
| 4905 | 1st | 170428 | 68 | 135 | 0.44444444 |
|  | last | 170910 | 70 |  |  |
| 4928 | 1st | 170420 | 65 | 145 | 0.62068966 |
|  | last | 170912 | 68 |  |  |
| 4920 | 1st | 161011 | 72 | 336 | 1.07142857 |
|  | last | 170912 | 84 |  |  |
| 4913 | 1st | 170624 | 69 | 78 | 0.96153846 |
|  | last | 170910 | 71.5 |  |  |
| 6045 | 1st | 170427 | 70 | 139 | 1.07913669 |
|  | last | 170913 | 75 |  |  |
| 4918 | 1st | 170910 | 75 | 78 | 0.19230769 |
|  | last | 171127 | 75.5 |  |  |
| 4951 | 1st | 161202 | 65 | 286 | 0.47202797 |
|  | last | 170914 | 69.5 |  |  |
| 6061 | 1st | 170912 | 70 | 78 | 1.15384615 |
|  | last | 171129 | 73 |  |  |
| 4934 | 1st | 160902 | 65.5 | 379 | 0.67282322 |
|  | last | 170916 | 74 |  |  |
| 4959 | 1st | 170916 | 72 | 72 | 1.25 |
|  | last | 171127 | 75 |  |  |
| 4971 | 1st | 170914 | 66 | 74 | 1.01351351 |
|  | last | 171127 | 68.5 |  |  |
| 4976 | 1st | 170912 | 71 | 77 | 1.75324675 |
|  | last | 171128 | 75.5 |  |  |
| 4952 | 1st | 170916 | 70.5 | 73 | 0.61643836 |
|  | last | 171128 | 72 |  |  |
| 4973 | 1st | 170912 | 73.5 | 77 | 0.1948052 |
|  | last | 171128 | 74 |  |  |
| 4989 | 1st | 170916 | 64.5 | 76 | 1.77631579 |
|  | last | 171201 | 69 |  |  |
| 4962 | 1st | 170914 | 60.5 | 78 | 1.73076923 |
|  | last | 171201 | 65 |  |  |
| 4991 | 1st | 170912 | 68 | 80 | 2.625 |
|  | last | 171201 | 75 |  |  |
| 4986 | 1st | 170912 | 72 | 80 | 1.5 |
|  | last | 171201 | 76 |  |  |
| 4968 | 1st | 170914 | 71.5 | 81 | 0.92592593 |
|  | last | 171204 | 74 |  |  |

Table S2. Observations (n=246) of juveniles with open or recently closed umbilical scar between April and May from 2013 to 2017.

|  |  | 2013 |  |  | 2014 |  |  | 2016 |  |  | 2017 |
| --- | --- | --- | --- | --- | --- | --- | --- | --- | --- | --- | --- |
| **Capture date** | **M(1) F (0)** | **TL (cm)** | **Capture date** | **M(1) F (0)** | **TL (cm)** | **Capture date** | **M(1) F (0)** | **TL (cm)** | **Capture date** | **M(1) F (0)** | **TL (cm)** |
| 130408 | 1 | 65 | 140418 | 1 | 68.5 | 160429 | 1 | 69 | 170404 | 0 | 69.5 |
| 130509 | 1 | 67 |  | 1 | 70.5 |  | 1 | 73.5 | 170408 | 0 | 89 |
| 130511 | 0 | 71 |  | 1 | 65 |  | 1 | 62 | 170411 | 1 | 81 |
|  | 0 | 61.5 |  | 0 | 69 |  | 0 | 70 | 170416 | 1 | 71 |
|  | 0 | 72 |  | 0 | 68.5 |  | 1 | 70 |  | 1 | 64 |
|  | 1 | 68.5 |  | 0 | 67 |  | 0 | 65 |  | 1 | 68 |
|  | 1 | 69 | 140419 | 0 | 68.5 |  | 0 | 69 | 170417 | 0 | 67 |
|  | 1 | 72 |  | 0 | 73.5 |  | 1 | 64.5 |  | 0 | 68 |
|  | 1 | 64 |  | 1 | 70.5 |  | 1 | 66.5 |  | 0 | 66 |
|  | 1 | 61.5 |  | 1 | 65 |  | 0 | 70 | 170419 | 1 | 65 |
| 130512 | 0 | 66 | 140420 | 1 | 67 |  | 1 | 66 |  | 1 | 68 |
|  | 0 | 71 |  | 0 | 73 | 160430 | 1 | 65.5 | 170420 | 1 | 65 |
|  | 1 | 69 |  | 0 | 69.5 |  | 1 | 68 |  | 0 | 70 |
| 130513 | 1 | 66 |  | 0 | 83 |  | 0 | 64 |  | 1 | 65 |
|  | 0 | 70.5 |  | 0 | 66 |  | 0 | 59 |  | 1 | 76 |
|  | 0 | 66.5 | 140421 | 1 | 72 |  | 1 | 86.5 |  | 1 | 78 |
|  | 0 | 69.9 |  | 1 | 71 | 160502 | 1 | 64 |  | 1 | 86 |
| 140418 | 1 | 68.5 |  | 1 | 72 |  | 1 | 70 | 170425 | 0 | 75 |
|  | 1 | 70.5 | 140422 | 0 | 66.5 |  | 1 | 67.5 | 170427 | 1 | 70 |
|  | 1 | 65 |  | 1 | 71.5 | 160503 | 1 | 65 |  | 1 | 61 |
|  | 0 | 69 |  | 0 | 71 | 160504 | 0 | 70 | 170428 | 1 | 68 |
|  | 0 | 68.5 |  | 0 | 94 |  | 0 | 76 |  | 1 | 69 |
|  | 0 | 67 |  | 1 | 68.5 |  | 0 | 76 | 170502 | 1 | 70 |
| 140419 | 0 | 68.5 | 140423 | 1 | 81.5 |  | 0 | 64 |  | 0 | 71 |
|  | 1 | 70.5 | 140523 | 1 | 74 |  | 0 | 68 | 170503 | 1 | 69 |
|  | 1 | 65 |  | 1 | 73 |  | 0 | 64.5 | 170504 | 0 | 69 |
| 140420 | 1 | 67 |  | 0 | 78.5 |  | 0 | 65.5 | 170506 | 1 | 67 |
|  | 0 | 69.5 |  | 1 | 69 | 160508 | 0 | 88 | 170507 | 0 | 70 |
|  | 0 | 66 |  | 1 | 65.5 |  | 1 | 58 |  |  |  |
| 140421 | 1 | 72 |  | 1 | 70 |  | 0 | 69 |  |  |  |
|  | 1 | 71 |  | 1 | 68 |  | 1 | 70 |  |  |  |
|  | 1 | 72 |  | 1 | 65 |  | 0 | 68 |  |  |  |
| 140422 | 0 | 66.5 |  | 0 | 71 | 160509 | 1 | 70 |  |  |  |
|  | 1 | 71.5 |  | 0 | 68.5 |  | 1 | 66 |  |  |  |
|  | 0 | 71 |  | 0 | 70.5 | 160510 | 1 | 59 |  |  |  |
|  | 1 | 68.5 |  | 1 | 69.5 |  |  |  |  |  |  |
| 140523 | 1 | 69 |  | 0 | 72 |  |  |  |  |  |  |
|  | 1 | 65.5 |  | 0 | 68 |  |  |  |  |  |  |
|  | 1 | 70 |  | 0 | 68.5 |  |  |  |  |  |  |
|  | 1 | 68 |  | 1 | 67 |  |  |  |  |  |  |
|  | 1 | 65 |  | 0 | 71 |  |  |  |  |  |  |
|  | 0 | 71 | 140524 | 0 | 70.5 |  |  |  |  |  |  |
|  | 0 | 68.5 | 140525 | 0 | 68.5 |  |  |  |  |  |  |
|  | 0 | 70.5 |  | 0 | 73 |  |  |  |  |  |  |
|  | 1 | 69.5 |  | 1 | 73 |  |  |  |  |  |  |
|  | 0 | 72 |  | 1 | 72 |  |  |  |  |  |  |
|  | 0 | 68 |  | 0 | 72 |  |  |  |  |  |  |
|  | 0 | 68.5 |  | 0 | 70 |  |  |  |  |  |  |
|  | 1 | 67 |  | 0 | 69 |  |  |  |  |  |  |
|  | 0 | 71 |  | 0 | 68.5 |  |  |  |  |  |  |
| 140524 | 0 | 70.5 |  | 0 | 66 |  |  |  |  |  |  |
| 140525 | 0 | 68.5 |  | 1 | 70 |  |  |  |  |  |  |
|  | 1 | 72 | 140527 | 0 | 68.5 |  |  |  |  |  |  |
|  | 0 | 72 | 140528 | 0 | 70 |  |  |  |  |  |  |
|  | 0 | 70 |  | 1 | 69 |  |  |  |  |  |  |
|  | 0 | 69 |  | 1 | 68 |  |  |  |  |  |  |
|  | 0 | 68.5 |  | 0 | 68.5 |  |  |  |  |  |  |
|  | 0 | 66 |  |  |  |  |  |  |  |  |  |
|  | 1 | 70 |  |  |  |  |  |  |  |  |  |
| 140527 | 0 | 68.5 |  |  |  |  |  |  |  |  |  |
| 140528 | 0 | 70 |  |  |  |  |  |  |  |  |  |
|  | 1 | 69 |  |  |  |  |  |  |  |  |  |
|  | 1 | 68 |  |  |  |  |  |  |  |  |  |
|  | 0 | 68.5 |  |  |  |  |  |  |  |  |  |
| 160429 | 1 | 69 |  |  |  |  |  |  |  |  |  |
|  | 1 | 62 |  |  |  |  |  |  |  |  |  |
|  | 0 | 70 |  |  |  |  |  |  |  |  |  |
|  | 1 | 70 |  |  |  |  |  |  |  |  |  |
|  | 0 | 65 |  |  |  |  |  |  |  |  |  |
|  | 0 | 69 |  |  |  |  |  |  |  |  |  |
|  | 1 | 64.5 |  |  |  |  |  |  |  |  |  |
|  | 1 | 66.5 |  |  |  |  |  |  |  |  |  |
|  | 0 | 70 |  |  |  |  |  |  |  |  |  |
|  | 1 | 66 |  |  |  |  |  |  |  |  |  |
| 160430 | 1 | 65.5 |  |  |  |  |  |  |  |  |  |
|  | 1 | 68 |  |  |  |  |  |  |  |  |  |
|  | 0 | 64 |  |  |  |  |  |  |  |  |  |
|  | 0 | 59 |  |  |  |  |  |  |  |  |  |
| 160502 | 1 | 64 |  |  |  |  |  |  |  |  |  |
|  | 1 | 70 |  |  |  |  |  |  |  |  |  |
|  | 1 | 67.5 |  |  |  |  |  |  |  |  |  |
| 160503 | 1 | 65 |  |  |  |  |  |  |  |  |  |
| 160504 | 0 | 70 |  |  |  |  |  |  |  |  |  |
|  | 0 | 64 |  |  |  |  |  |  |  |  |  |
|  | 0 | 68 |  |  |  |  |  |  |  |  |  |
|  | 0 | 64.5 |  |  |  |  |  |  |  |  |  |
|  | 0 | 65.5 |  |  |  |  |  |  |  |  |  |
| 160508 | 1 | 58 |  |  |  |  |  |  |  |  |  |
|  | 0 | 69 |  |  |  |  |  |  |  |  |  |
|  | 1 | 70 |  |  |  |  |  |  |  |  |  |
|  | 0 | 68 |  |  |  |  |  |  |  |  |  |
| 160509 | 1 | 70 |  |  |  |  |  |  |  |  |  |
|  | 1 | 66 |  |  |  |  |  |  |  |  |  |
| 160510 | 1 | 59 |  |  |  |  |  |  |  |  |  |
| 170404 | 0 | 69.5 |  |  |  |  |  |  |  |  |  |
| 170416 | 1 | 71 |  |  |  |  |  |  |  |  |  |
|  | 1 | 64 |  |  |  |  |  |  |  |  |  |
|  | 1 | 68 |  |  |  |  |  |  |  |  |  |
| 170417 | 0 | 67 |  |  |  |  |  |  |  |  |  |
|  | 0 | 68 |  |  |  |  |  |  |  |  |  |
|  | 0 | 66 |  |  |  |  |  |  |  |  |  |
| 170419 | 1 | 65 |  |  |  |  |  |  |  |  |  |
|  | 1 | 68 |  |  |  |  |  |  |  |  |  |
| 170420 | 1 | 65 |  |  |  |  |  |  |  |  |  |
|  | 0 | 70 |  |  |  |  |  |  |  |  |  |
|  | 1 | 65 |  |  |  |  |  |  |  |  |  |
| 170427 | 1 | 70 |  |  |  |  |  |  |  |  |  |
|  | 1 | 61 |  |  |  |  |  |  |  |  |  |
| 170428 | 1 | 68 |  |  |  |  |  |  |  |  |  |
|  | 1 | 69 |  |  |  |  |  |  |  |  |  |
| 170502 | 1 | 70 |  |  |  |  |  |  |  |  |  |
|  | 0 | 71 |  |  |  |  |  |  |  |  |  |
| 170503 | 1 | 69 |  |  |  |  |  |  |  |  |  |
| 170504 | 0 | 69 |  |  |  |  |  |  |  |  |  |
| 170506 | 1 | 67 |  |  |  |  |  |  |  |  |  |
| 170507 | 0 | 70 |  |  |  |  |  |  |  |  |  |

Table S3 Genetic diversity of 9 microsatellite loci in 5 cohorts.

| Locus name | Pop(N) | Ho | He |
| --- | --- | --- | --- |
| LS11 | 2013(10) | 0.80 | 0.84 |
| LS11 | 2014(24) | 0.83 | 0.84 |
| LS11 | 2015(85) | 0.65 | 0.84 |
| LS11 | 2016(45) | 0.62 | 0.82 |
| LS11 | 2017(24) | 0.67 | 0.82 |
| LS24 | 2013(10) | 0.20 | 0.19 |
| LS24 | 2014(24) | 0.29 | 0.38 |
| LS24 | 2015(85) | 0.35 | 0.40 |
| LS24 | 2016(45) | 0.33 | 0.38 |
| LS24 | 2017(24) | 0.42 | 0.35 |
| LS54 | 2013(10) | 0.40 | 0.35 |
| LS54 | 2014(24) | 0.54 | 0.46 |
| LS54 | 2015(85) | 0.42 | 0.41 |
| LS54 | 2016(45) | 0.40 | 0.34 |
| LS54 | 2017(24) | 0.38 | 0.33 |
| CP190 | 2013(10) | 0.50 | 0.75 |
| CP190 | 2014(24) | 0.42 | 0.67 |
| CP190 | 2015(85) | 0.42 | 0.65 |
| CP190 | 2016(45) | 0.44 | 0.68 |
| CP190 | 2017(24) | 0.38 | 0.72 |
| NA08 | 2013(10) | 0.90 | 0.89 |
| NA08 | 2014(24) | 0.58 | 0.85 |
| NA08 | 2015(85) | 0.72 | 0.88 |
| NA08 | 2016(45) | 0.84 | 0.89 |
| NA08 | 2017(24) | 0.75 | 0.90 |
| NA10 | 2013(10) | 0.50 | 0.65 |
| NA10 | 2014(24) | 0.48 | 0.67 |
| NA10 | 2015(85) | 0.61 | 0.66 |
| NA10 | 2016(45) | 0.67 | 0.68 |
| NA10 | 2017(24) | 0.67 | 0.66 |
| NA12 | 2013(10) | 0.33 | 0.39 |
| NA12 | 2014(24) | 0.12 | 0.53 |
| NA12 | 2015(85) | 0.42 | 0.41 |
| NA12 | 2016(45) | 0.44 | 0.41 |
| NA12 | 2017(24) | 0.33 | 0.51 |
| NA13 | 2013(10) | 0.70 | 0.82 |
| NA13 | 2014(24) | 0.67 | 0.80 |
| NA13 | 2015(85) | 0.48 | 0.80 |
| NA13 | 2016(45) | 0.67 | 0.84 |
| NA13 | 2017(24) | 0.83 | 0.88 |
| NA14 | 2013(10) | 0.60 | 0.79 |
| NA14 | 2014(24) | 0.65 | 0.89 |
| NA14 | 2015(85) | 0.63 | 0.88 |
| NA14 | 2016(45) | 0.64 | 0.89 |
| NA14 | 2017(24) | 0.50 | 0.90 |
| NA16 | 2013(10) | 0.50 | 0.85 |
| NA16 | 2014(24) | 0.58 | 0.77 |
| NA16 | 2015(85) | 0.61 | 0.80 |
| NA16 | 2016(45) | 0.58 | 0.83 |
| NA16 | 2017(24) | 0.75 | 0.81 |
| NA19 | 2013(10) | 0.00 | 0.19 |
| NA19 | 2014(24) | 0.00 | 0.34 |
| NA19 | 2015(85) | 0.01 | 0.33 |
| NA19 | 2016(45) | 0.05 | 0.39 |
| NA19 | 2017(24) | 0.00 | 0.34 |
| NA20 | 2013(10) | 0.20 | 0.19 |
| NA20 | 2014(24) | 0.29 | 0.27 |
| NA20 | 2015(85) | 0.20 | 0.19 |
| NA20 | 2016(45) | 0.23 | 0.21 |
| NA20 | 2017(24) | 0.25 | 0.23 |

Table S4. Results of cohort assignment.

| Sample No. | TL(cm) | Weight(kg) | Female(0) Male (1) | Date of capture | Age by month (y=0.868 x+67.44) | Cohort | Capture site |
| --- | --- | --- | --- | --- | --- | --- | --- |
| 1 | 90 | 3.45 | 1 | 2015/04/11 | 25.99078341 | 2013 | North Coast |
| 2 | 88 | 3.69 | 0 | 2015/04/11 | 23.68663594 | 2013 | Lagoon mouth |
| 3 | 82 | 2.5 | 0 | 2015/04/12 | 16.77419355 | 2013 | Lagoon mouth |
| 4 | 62.5 | 1.135 | 1 | 2015/04/13 | -5.69124424 | 2015 | Lagoon mouth |
| 5 | 79 | 2.33 | 0 | 2015/04/14 | 13.31797235 | 2014 | Lagoon mouth |
| 6 | 82.5 | 2.85 | 1 | 2015/04/15 | 17.35023041 | 2013 | Lagoon mouth |
| 7 | 69.5 | 1.775 | 1 | 2015/05/16 | 2.373271889 | 2015 | Lagoon |
| 8 | 69.5 | 1.665 | 0 | 2015/05/16 | 2.373271889 | 2015 | Lagoon |
| 9 | 84 | 3.08 | 1 | 2015/05/16 | 19.07834101 | 2013 | Lagoon |
| 10 | 69.5 | 1.54 | 1 | 2015/05/17 | 2.373271889 | 2015 | Lagoon |
| 11 | 69.5 | 1.565 | 1 | 2015/05/17 | 2.373271889 | 2015 | Lagoon |
| 12 | 71.5 | 1.855 | 0 | 2015/05/17 | 4.677419355 | 2015 | Lagoon |
| 13 | 65.5 | 1.455 | 1 | 2015/05/17 | -2.235023041 | 2015 | North Coast |
| 14 | 69 | 1.685 | 1 | 2015/05/18 | 1.797235023 | 2015 | Lagoon |
| 15 | 67.5 | 1.455 | 1 | 2015/05/18 | 0.069124424 | 2015 | Lagoon |
| 16 | 66.5 | 1.54 | 0 | 2015/05/18 | -1.082949309 | 2015 | North Coast |
| 17 | 85 | 2.875 | 1 | 2015/05/18 | 20.23041475 | 2013 | Lagoon mouth |
| 18 | 69.5 | 1.615 | 1 | 2015/05/18 | 2.373271889 | 2015 | Lagoon mouth |
| 19 | 70 | 1.635 | 1 | 2015/05/18 | 2.949308756 | 2015 | Lagoon mouth |
| 20 | 67 | 1.405 | 1 | 2015/05/18 | -0.506912442 | 2015 | Lagoon mouth |
| 21 | 68.5 | 1.87 | 0 | 2015/05/18 | 1.221198157 | 2015 | North Coast |
| 22 | 79.5 | 2.36 | 0 | 2015/05/18 | 13.89400922 | 2014 | North Coast |
| 23 | 69.5 | 1.455 | 1 | 2015/05/19 | 2.373271889 | 2015 | North Coast |
| 24 | 71 | 1.94 | 0 | 2015/05/19 | 4.101382488 | 2015 | North Coast |
| 25 | 70.5 | 1.75 | 0 | 2015/05/19 | 3.525345622 | 2015 | North Coast |
| 26 | 70 | 1.875 | 1 | 2015/05/19 | 2.949308756 | 2015 | North Coast |
| 27 | 72 | 1.795 | 0 | 2015/05/19 | 5.253456221 | 2015 | North Coast |
| 28 | 73.5 | 1.7 | 1 | 2015/05/19 | 6.98156682 | 2014 | North Coast |
| 29 | 72.5 | 1.835 | 0 | 2015/05/19 | 5.829493088 | 2014 | North Coast |
| 30 | 66 | 1.33 | 0 | 2015/05/19 | -1.658986175 | 2015 | North Coast |
| 31 | 78 | 2.18 | 0 | 2015/05/19 | 12.16589862 | 2014 | North Coast |
| 32 | 68 | 1.775 | 0 | 2015/05/20 | 0.64516129 | 2015 | North Coast |
| 33 | 71.5 | 1.915 | 1 | 2015/05/20 | 4.677419355 | 2015 | North Coast |
| 34 | 68.5 | 1.485 | 1 | 2015/05/23 | 1.221198157 | 2015 | North Coast |
| 35 | 68 | 1.64 | 0 | 2015/05/23 | 0.64516129 | 2015 | North Coast |
| 36 | 67 | 1.38 | 1 | 2015/05/23 | -0.506912442 | 2015 | North Coast |
| 37 | 69.5 | 1.72 | 1 | 2015/05/23 | 2.373271889 | 2015 | North Coast |
| 38 | 71 | 1.56 | 0 | 2015/05/24 | 4.101382488 | 2015 | North Coast |
| 39 | 67.5 | 1.54 | 1 | 2015/05/24 | 0.069124424 | 2015 | North Coast |
| 40 | 69.5 | 1.68 | 0 | 2015/05/24 | 2.373271889 | 2015 | North Coast |
| 41 | 67.5 | 1.595 | 1 | 2015/05/24 | 0.069124424 | 2015 | North Coast |
| 42 | 81.5 | 2.775 | 1 | 2015/05/25 | 16.19815668 | 2014 | North Coast |
| 43 | 67.5 | 1.54 | 0 | 2015/05/25 | 0.069124424 | 2015 | North Coast |
| 44 | 78.5 | 2.415 | 0 | 2015/07/17 | 12.74193548 | 2014 | Lagoon |
| 45 | 82 | 2.825 | 1 | 2015/07/17 | 16.77419355 | 2014 | Lagoon |
| 46 | 70.5 | 1.93 | 1 | 2015/07/17 | 3.525345622 | 2015 | Lagoon |
| 47 | 73 | 1.62 | 1 | 2015/07/17 | 6.405529954 | 2015 | Lagoon mouth |
| 48 | 72.5 | 1.795 | 1 | 2015/07/17 | 5.829493088 | 2015 | Lagoon mouth |
| 49 | 69 | 1.505 | 0 | 2015/07/18 | 1.797235023 | 2015 | North Coast |
| 50 | 76 | 2.055 | 0 | 2015/07/18 | 9.861751152 | 2014 | Lagoon |
| 51 | 71.5 | 1.68 | 0 | 2015/07/18 | 4.677419355 | 2015 | Lagoon |
| 52 | 68 | 1.38 | 1 | 2015/07/18 | 0.64516129 | 2015 | Lagoon |
| 53 | 74.5 | 1.935 | 1 | 2015/07/18 | 8.133640553 | 2014 | Lagoon mouth |
| 54 | 75 | 1.795 | 1 | 2015/07/18 | 8.709677419 | 2014 | Lagoon mouth |
| 55 | 69.5 | 1.62 | 1 | 2015/07/18 | 2.373271889 | 2015 | North Coast |
| 56 | 75 | 1.985 | 0 | 2015/07/19 | 8.709677419 | 2014 | North Coast |
| 57 | 73 | 1.766 | 0 | 2015/07/19 | 6.405529954 | 2015 | North Coast |
| 58 | 71.5 | 1.365 | 1 | 2015/07/19 | 4.677419355 | 2015 | North Coast |
| 59 | 75.5 | 1.535 | 1 | 2015/07/19 | 9.285714286 | 2014 | North Coast |
| 60 | 72 | 1.735 | 1 | 2015/07/19 | 5.253456221 | 2015 | North Coast |
| 61 | 73 | 1.75 | 1 | 2015/07/19 | 6.405529954 | 2015 | Lagoon mouth |
| 63 | 70.5 | 1.66 | 1 | 2015/07/20 | 3.525345622 | 2015 | North Coast |
| 64 | 77.5 | 2.11 | 1 | 2015/07/21 | 11.58986175 | 2014 | North Coast |
| 65 | 71.5 | 1.475 | 0 | 2015/07/21 | 4.677419355 | 2015 | Lagoon mouth |
| 66 | 72.5 | 1.74 | 1 | 2015/07/22 | 5.829493088 | 2015 | Lagoon mouth |
| 67 | 71 | 1.635 | 0 | 2015/07/22 | 4.101382488 | 2015 | Lagoon mouth |
| 69 | 73.2 | 1.63 | 1 | 2015/10/09 | 6.6359447 | 2015 | Lagoon |
| 70 | 71 | 1.49 | 0 | 2015/10/09 | 4.101382488 | 2015 | North Coast |
| 71 | 71.5 | 1.715 | 0 | 2015/10/11 | 4.677419355 | 2015 | North Coast |
| 72 | 73.5 | 1.945 | 1 | 2015/10/11 | 6.98156682 | 2015 | North Coast |
| 73 | 71 | 1.845 | 0 | 2015/10/11 | 4.101382488 | 2015 | North Coast |
| 74 | 76 | 2.235 | 0 | 2015/10/12 | 9.861751152 | 2014 | North Coast |
| 75 | 71 | 1.55 | 0 | 2015/10/12 | 4.101382488 | 2015 | Lagoon mouth |
| 76 | 73 | 1.91 | 0 | 2015/10/13 | 6.405529954 | 2015 | North Coast |
| 77 | 101 | 5.385 | 0 | 2015/10/13 | 38.66359447 | 2012 | North Coast |
| 78 | 76.5 | 2.03 | 1 | 2015/10/13 | 10.43778802 | 2014 | Lagoon mouth |
| 79 | 72 | 1.6 | 1 | 2015/10/13 | 5.253456221 | 2015 | Lagoon mouth |
| 80 | 95 | 3.625 | 1 | 2015/10/13 | 31.75115207 | 2013 | Lagoon mouth |
| 81 | 64 | 1.34 | 0 | 2015/10/13 | -3.963133641 | 2015 | Lagoon mouth |
| 82 | 82 | 3.3 | 1 | 2015/10/14 | 16.77419355 | 2014 | North Coast |
| 83 | 80 | 3.4 | 1 | 2015/10/14 | 14.47004608 | 2014 | North Coast |
| 84 | 73.5 | 2.6 | 1 | 2015/10/14 | 6.98156682 | 2015 | North Coast |
| 85 | 61.5 | 1.4 | 0 | 2015/10/14 | -6.843317972 | 2015 | Lagoon mouth |
| 86 | 71 | 2.3 | 0 | 2015/10/14 | 4.101382488 | 2015 | Lagoon mouth |
| 87 | 74.5 | 2.4 | 1 | 2015/10/14 | 8.133640553 | 2015 | Lagoon mouth |
| 88 | 91.5 | 3.565 | 1 | 2015/10/14 | 27.71889401 | 2013 | North Coast |
| 89 | 71.5 | 1.335 | 1 | 2015/11/6 | 4.677419355 | 2015 | Lagoon |
| 90 | 82.5 | 2.475 | 1 | 2015/11/6 | 17.35023041 | 2014 | Lagoon |
| 91 | 69 | 1.575 | 1 | 2015/11/6 | 1.797235023 | 2015 | Lagoon mouth |
| 92 | 73 | 1.905 | 1 | 2015/11/6 | 6.405529954 | 2015 | Lagoon mouth |
| 93 | 66 | 1.505 | 1 | 2015/11/6 | -1.658986175 | 2015 | Lagoon mouth |
| 94 | 71 | 1.645 | 1 | 2015/11/6 | 4.101382488 | 2015 | Lagoon mouth |
| 95 | 79 | 1.88 | 0 | 2015/11/6 | 13.31797235 | 2014 | Lagoon mouth |
| 96 | 78 | 2.145 | 1 | 2015/11/6 | 12.16589862 | 2014 | Lagoon mouth |
| 98 | 53 | 0.765 | 0 | 2015/11/6 | -16.6359447 | 2015 | Lagoon mouth |
| 99 | 74 | 1.92 | 1 | 2015/11/6 | 7.557603687 | 2015 | Lagoon |
| 100 | 74 | 2.065 | 0 | 2015/11/6 | 7.557603687 | 2015 | Lagoon |
| 101 | 76.5 | 1.965 | 0 | 2015/11/6 | 10.43778802 | 2015 | Lagoon |
| 102 | 75.5 | 1.855 | 1 | 2015/11/7 | 9.285714286 | 2015 | Lagoon mouth |
| 103 | 66.5 | 1.42 | 1 | 2015/11/7 | -1.082949309 | 2015 | Lagoon mouth |
| 104 | 74.5 | 1.655 | 1 | 2015/11/7 | 8.133640553 | 2015 | Lagoon mouth |
| 106 | 91 | 3.48 | 1 | 2015/11/8 | 27.14285714 | 2013 | North Coast |
| 107 | 76.5 | 1.65 | 0 | 2015/11/8 | 10.43778802 | 2015 | Lagoon mouth |
| 108 | 75.5 | 1.69 | 1 | 2015/11/8 | 9.285714286 | 2015 | Lagoon mouth |
| 109 | 74.5 | 1.675 | 0 | 2015/11/8 | 8.133640553 | 2015 | Lagoon mouth |
| 111 | 70 | 1.065 | 1 | 2015/11/9 | 2.949308756 | 2015 | Lagoon |
| 112 | 76.4 | 2.160 | 1 | 2015/11/9 | 10.32258065 | 2015 | Lagoon |
| 113 | 69 | 1.735 | 1 | 2016/04/29 | 1.797235023 | 2016 | Lagoon mouth |
| 114 | 73.5 | 1.54 | 1 | 2016/04/29 | 6.98156682 | 2015 | Lagoon |
| 115 | 62 | 1.32 | 1 | 2016/04/29 | -6.267281106 | 2016 | Lagoon mouth |
| 116 | 70 | 1.575 | 0 | 2016/04/29 | 2.949308756 | 2016 | Lagoon mouth |
| 117 | 70 | 1.79 | 1 | 2016/04/29 | 2.949308756 | 2016 | Lagoon mouth |
| 118 | 65 | 1.44 | 0 | 2016/04/29 | -2.811059908 | 2016 | Lagoon mouth |
| 119 | 69 | 1.68 | 0 | 2016/04/29 | 1.797235023 | 2016 | Lagoon mouth |
| 120 | 64.5 | 1.57 | 1 | 2016/04/29 | -3.387096774 | 2016 | Lagoon mouth |
| 121 | 66.5 | 1.6 | 1 | 2016/04/29 | -1.082949309 | 2016 | Lagoon mouth |
| 122 | 70 | 1.72 | 0 | 2016/04/29 | 2.949308756 | 2016 | Lagoon mouth |
| 123 | 66 | 1.5 | 1 | 2016/04/29 | -1.658986175 | 2016 | Lagoon mouth |
| 124 | 65.5 | 1.165 | 1 | 2016/04/30 | -2.235023041 | 2016 | Lagoon |
| 125 | 68 | 1.795 | 1 | 2016/04/30 | 0.64516129 | 2016 | Lagoon mouth |
| 126 | 64 | 1.25 | 0 | 2016/04/30 | -3.963133641 | 2016 | Lagoon mouth |
| 127 | 59 | 0.89 | 0 | 2016/04/30 | -9.723502304 | 2016 | Lagoon mouth |
| 128 | 86.5 | 2.885 | 1 | 2016/04/30 | 21.95852535 | 2014 | Lagoon mouth |
| 129 | 64 | 1.34 | 1 | 2016/05/02 | -3.963133641 | 2016 | Lagoon mouth |
| 130 | 70 | 1.755 | 1 | 2016/05/02 | 2.949308756 | 2016 | Lagoon |
| 131 | 67.5 | 1.7 | 1 | 2016/05/02 | 0.069124424 | 2016 | Lagoon mouth |
| 132 | 65 | 1.44 | 1 | 2016/05/03 | -2.811059908 | 2016 | Lagoon |
| 133 | 70 | 1.745 | 0 | 2016/05/04 | 2.949308756 | 2016 | North Coast |
| 134 | 76 | 1.885 | 0 | 2016/05/04 | 9.861751152 | 2015 | Lagoon |
| 135 | 76 | 2.015 | 0 | 2016/05/04 | 9.861751152 | 2015 | Lagoon |
| 136 | 64 | 1.405 | 0 | 2016/05/04 | -3.963133641 | 2016 | Lagoon |
| 137 | 68 | 1.605 | 0 | 2016/05/04 | 0.64516129 | 2016 | Lagoon mouth |
| 138 | 64.5 | 1.275 | 0 | 2016/05/04 | -3.387096774 | 2016 | Lagoon mouth |
| 139 | 65.5 | 1.49 | 0 | 2016/05/04 | -2.235023041 | 2016 | Lagoon mouth |
| 140 | 58 | 1.055 | 1 | 2016/05/08 | -10.87557604 | 2016 | Lagoon mouth |
| 141 | 69 | 1.56 | 0 | 2016/05/08 | 1.797235023 | 2016 | Lagoon mouth |
| 142 | 70 | 1.675 | 1 | 2016/05/08 | 2.949308756 | 2016 | Lagoon mouth |
| 143 | 68 | 1.52 | 0 | 2016/05/08 | 0.64516129 | 2016 | Lagoon mouth |
| 144 | 88 | 3.5 | 0 | 2016/05/08 | 23.68663594 | 2014 | Lagoon mouth |
| 145 | 70 | 1.78 | 1 | 2016/05/09 | 2.949308756 | 2016 | Lagoon |
| 146 | 66 | 1.51 | 1 | 2016/05/09 | -1.658986175 | 2016 | North Coast |
| 147 | 59 | 1.02 | 1 | 2016/05/10 | -9.723502304 | 2016 | Lagoon |
| 148 | 91.5 | 3.97 | 0 | 2015/05/19 | 27.71889401 | 2013 | North Coast |
| 149 | 70.5 | 1.655 | 0 | 2015/05/16 | 3.525345622 | 2015 | Lagoon |
| 150 | 66.5 | 1.495 | 1 | 2015/05/16 | -1.082949309 | 2015 | Lagoon mouth |
| 151 | 65 | 1.275 | 1 | 2015/05/16 | -2.811059908 | 2015 | Lagoon mouth |
| 152 | 66 | 1.315 | 1 | 2015/05/17 | -1.658986175 | 2015 | Lagoon mouth |
| 545 | 73 | 1.695 | 1 | 2017/01/14 | 6.405529954 | 2016 | Lagoon mouth |
| 546 | 65 | 1.195 | 1 | 2017/01/14 | -2.811059908 | 2017 | Lagoon mouth |
| 547 | 63 | 1.905 | 1 | 2017/01/14 | -5.115207373 | 2017 | Lagoon mouth |
| 548 | 68.5 | 1.420 | 0 | 2017/01/15 | 1.221198157 | 2016 | Lagoon mouth |
| 549 | 72 | 1.700 | 0 | 2017/01/15 | 5.253456221 | 2016 | Lagoon mouth |
| 550 | 70 | 1.685 | 0 | 2017/01/15 | 2.949308756 | 2016 | Lagoon mouth |
| 551 | 69.5 | 1.650 | 1 | 2017/01/15 | 2.373271889 | 2016 | Lagoon mouth |
| 552 | 67.5 | 1.405 | 1 | 2017/01/15 | 0.069124424 | 2016 | Lagoon mouth |
| 553 | 69 | 1.480 | 1 | 2017/01/15 | 1.797235023 | 2016 | Lagoon mouth |
| 554 | 65 | 1.155 | 1 | 2017/01/15 | -2.811059908 | 2017 | Lagoon mouth |
| 555 | 74 | 2.120 | 0 | 2017/01/15 | 7.557603687 | 2016 | Lagoon mouth |
| 556 | 64.5 | 1.050 | 0 | 2017/01/15 | -3.387096774 | 2017 | Lagoon mouth |
| 557 | 62.5 | 1.060 | 1 | 2017/01/15 | -5.69124424 | 2017 | Lagoon mouth |
| 558 | 68 | 1.545 | 1 | 2017/01/16 | 0.64516129 | 2016 | Lagoon mouth |
| 559 | 66.5 | 1.180 | 1 | 2017/01/16 | -1.082949309 | 2017 | North Coast |
| 560 | 70 | 2.2 | 1 | 2017/01/16 | 2.949308756 | 2016 | North Coast |
| 561 | 96 | 3.62 | 0 | 2017/01/17 | 32.90322581 | 2014 | North Coast |
| 566 | 69.5 | 1.605 | 0 | 2017/4/4 | 2.373271889 | 2017 | Lagoon |
| 567 | 89 | 2.71 | 0 | 2017/4/8 | 24.83870968 | 2015 | Lagoon mouth |
| 568 | 81 | 2.165 | 1 | 2017/4/11 | 15.62211982 | 2016 | Lagoon mouth |
| 569 | 71 | 1.675 | 1 | 2017/4/16 | 4.101382488 | 2016 | Lagoon |
| 570 | 64 | 1.33 | 1 | 2017/4/16 | -3.963133641 | 2017 | Lagoon |
| 571 | 68 | 1.765 | 1 | 2017/4/16 | 0.64516129 | 2017 | Lagoon |
| 572 | 67 | 1.67 | 0 | 2017/4/17 | -0.506912442 | 2017 | Lagoon |
| 573 | 68 | 1.39 | 0 | 2017/4/17 | 0.64516129 | 2017 | Lagoon |
| 574 | 66 | 1.37 | 0 | 2017/4/17 | -1.658986175 | 2017 | Lagoon |
| 575 | 65 | 1.36 | 1 | 2017/4/19 | -2.811059908 | 2017 | Lagoon mouth |
| 576 | 68 | 1.65 | 1 | 2017/4/19 | 0.64516129 | 2017 | Lagoon mouth |
| 577 | 65 | 1.41 | 1 | 2017/4/20 | -2.811059908 | 2017 | North Coast |
| 578 | 70 | 1.82 | 0 | 2017/4/20 | 2.949308756 | 2017 | Lagoon |
| 579 | 65 | 1.33 | 1 | 2017/4/20 | -2.811059908 | 2017 | Lagoon |
| 580 | 76 | 2.485 | 1 | 2017/4/20 | 9.861751152 | 2016 | North Coast |
| 581 | 78 | 2.345 | 1 | 2017/4/20 | 12.16589862 | 2016 | North Coast |
| 583 | 75 | 1.84 | 0 | 2017/4/25 | 8.709677419 | 2016 | North Coast |
| 585 | 70 | 1.8 | 1 | 2017/4/27 | 2.949308756 | 2017 | North Coast |
| 586 | 61 | 1.13 | 1 | 2017/4/27 | -7.419354839 | 2017 | Lagoon |
| 587 | 68 | 1.7 | 1 | 2017/4/28 | 0.64516129 | 2017 | Lagoon |
| 588 | 69 | 1.65 | 1 | 2017/4/28 | 1.797235023 | 2017 | Lagoon |
| 589 | 70 | 1.64 | 1 | 2017/5/2 | 2.949308756 | 2017 | Lagoon |
| 590 | 71 | 1.6 | 0 | 2017/5/2 | 4.101382488 | 2017 | Lagoon |
| 591 | 69 | 1.35 | 1 | 2017/53 | 1.797235023 | 2017 | Lagoon mouth |

Table S5 Pairwise Fst values among 5 cohorts (below diagonal), p value (above diagonal).

|  | 2013 | 2014 | 2015 | 2016 | 2017 |
| --- | --- | --- | --- | --- | --- |
| 2013 | - | n.s. | n.s. | n.s. | n.s. |
| 2014 | -0.008513 | - | n.s. | n.s. | n.s. |
| 2015 | 0.00436 | -0.003876 | - | n.s. | n.s. |
| 2016 | 0.000473 | 0.001511 | 0.001401 | - | n.s. |
| 2017 | -0.004188 | -0.003166 | -0.000732 | -0.007917 | - |

Table S6 Pairwise Fst values among 3 sampling sites (below diagonal), p value (above diagonal).

|  | North | Lagoon mouth | Lagoon |
| --- | --- | --- | --- |
| North | - | n.s. | n.s. |
| Lagoon mouth | 0.002039 | - | n.s. |
| Lagoon | 0.002075 | 0.004534 | - |
